# Modelling Dose Effects from Space Irradiations: Combination of High-LET and Low-LET Radiations with a Modified Microdosimetric Kinetic Model

**DOI:** 10.3390/life10090161

**Published:** 2020-08-23

**Authors:** Alejandro Bertolet, Alejandro Carabe

**Affiliations:** Department of Radiation Oncology, Hospital of the University of Pennsylvania, Philadelphia, PA 19104, USA; alejandro.bertoletreina@pennmedicine.upenn.edu

**Keywords:** radiation mixing, ion therapy, MKM, LET

## Abstract

The Microdosimetric Kinetic Model (MKM) to predict the effects of ionizing radiation on cell colonies is studied and reformulated for the case of high-linear energy transfer (LET) radiations with a low dose. When the number of radiation events happening in a subnuclear domain follows a Poisson distribution, the MKM predicts a linear-quadratic (LQ) survival curve. We show that when few events occur, as for high-LET radiations at doses lower than the mean specific energy imparted to the nucleus, $z_{F,n}$, a Poisson distribution can no longer be assumed and an initial pure linear relationship between dose and survival fraction should be observed. Predictions of survival curves for combinations of high-LET and low-LET radiations are produced under two assumptions for their comparison: independent and combined action. Survival curves from previously published articles of V79 cell colonies exposed to X-rays, α particles, Ar-ions, Fe-ions, Ne-ions and mixtures of X-rays and each one of the ions are predicted according to the modified MKM. We conclude that mixtures of high-LET and low-LET radiations may enhance the effect of individual actions due to the increase of events in domains provided by the low-LET radiation. This hypothesis is only partially validated by the analyzed experiments.

## 1. Introduction

Ionizing radiations are capable of compromising the viability of live organisms and the functionality of organs and tissues. Their effect is largely related to the absorbed dose, which is defined as the energy per unit mass imparted to a certain volume. According to the Linear No-Threshold Relationship, even very low doses are capable of originating carcinogenic events with a probability proportional to the absorbed dose, although this assumption has been challenged [1,2]. As doses increase, the effects become deterministic, meaning that cumulative cellular damage or even death will occur at larger doses, mainly due to damage to the DNA in the cell nuclei. However, not all radiations have the same pattern of local energy deposition, meaning that different radiations do not produce equal effects for the same dose. For example, heavy ions tend to locally concentrate their interactions in reduced volumes, leading to clustered damage to the DNA [3]. In contrast, X-rays generally transfer energy in a more spread way, producing more isolated damages to the DNA, which are more easily repairable [4]. Linear energy transfer (LET) is a physical quantity defined as the amount of energy imparted in electronic collisions per unit track length of the considered radiation [5,6], thus representing a measure of the local concentration of energy. Therefore, a relation can be established between LET and the biological effectiveness of a radiation for a given dose.

In order to predict biological outcomes of expositions to radiation, several theories and models have been developed. In particular, the theory of dual radiation action (TDRA) [7,8] states that radiation is able to produce lethal and sublethal lesions to cells, and that sublethal lesions can interact in pairs to induce lethal damage. This assumption may lead to the more general Linear Quadratic (LQ) model, in which the lethality among a set of cells exposed to radiation follows a Poisson distribution whose mean depends on the dose with a linear and a quadratic component, related to single-track and two-track events, respectively, although the interpretation of the quadratic term is still under debate [9]. Although the LQ model is widely accepted, its validity is limited, and other models can be alternatively considered [10]. In particular, the repair-misrepair-fixation (RMF) [11], the giant loop binary lesion (GLOBLE) [12] or the biophysical analysis of cell death and chromosome aberrations (BIANCA) [13] are alternative descriptions of the interaction among lesions provoked by the DNA, including the mechanisms of repair of the cell or the dose rate effects. Other models are used specifically to include the dependence of the biological response on the LET, or more generally, the local concentration of energy, such as the Local Effect Model (LEM) [14,15] or the Microdosimetric Kinetic Model (MKM) [16,17]. The original MKM has also been modified to include saturation-corrected parameters [18,19], as well as inter-cell signalization effects [20].

These two models are currently used in clinical radiotherapy with carbon ions [21,22]. In particular, the MKM relies on the TDRA and assumes that the microscopic pattern of energy deposition leads the particular probability of a cell to survive to an arrest of radiation. In this sense, the MKM predicts that the linear component of the lethality probability is supposed to be enhanced as depositions become more locally concentrated, while the quadratic component remains unaffected. However, experimental survival curves for high-LET radiations seem to approach to a purely linear form [23,24,25] not predicted by the MKM. 

In this work, we explore the principles of the MKM to determine under what conditions survival curves after expositions to radiation tend to be linear, in particular when the number of radiation tracks involved in the dose deposition is low, excluding non-targeted effects [20,26]. This can lead to a study of the combined effect of mixed radiations. This is of great importance for applications in space, where astronauts are simultaneously exposed to combinations of radiations of different LET [23]. When combining two radiations or, in general, effectors, whether the combined result can be explained as the superposition of the individual effects or it is greater, the combination is called additive or synergistic [27]. Although some experimental studies point towards a possible synergistic effect when combining high-LET and low-LET radiations [24,25,28,29], the evidence is limited, and other studies indicate contradictory results [30,31,32,33,34]. This work reviews some of those works and compares the predictions after considering the effects of a low number of incident radiation tracks.

## 2. Materials and Methods

### 2.1. Low-Dose Region in the MKM

The MKM relies on the concept of domain, which is intended to represent a structure in which two sublethal lesions can combine in pairs to produce a lethal lesion. As the MKM assumes domains with spherical shape, the radius of the domain can be understood as the maximum distance at which a sublethal lesion can interact with another to form a lethal lesion. An event in a domain contains all the interactions between an incident particle and the DNA mass contained in that domain, including all the energy depositions (ionizations and excitations) by the primary particle and all the energy depositions by the δ rays generated along by the primary particle. A given radiation, specified by the type of the particle, i.e., mass and charge, and its kinetic energy stochastically interact with the domain depositing a specific energy $z_{1}$ in a single event, following a probability distribution $f\left(z_{1}\right)$. The mean—also called frequency average—of this distribution is notated by $z_{F}$, its variance as $V_{z_{1}}$, while the dose average of this distribution is defined as $z_{D}=\bar{z_{1}^{2}}/z_{F}$. Solving the microdosimetric kinetic equations as done by Hawkins, ref. [16] the mean number of lethal lesions in the domains receiving dose z, $\langle L_{d}|z\rangle$, is given by

(1) $$\langle L_{d}|z\rangle =A\langle g\rangle z+B\langle g^{2}\rangle z^{2}$$

where A and B are functions of the proportionality constants for the processes proposed in the model, g is the mass of DNA contained in each domain and 〈g〉 represents its average among domains. The mean number of events in a domain is $\mu =D/z_{F}$. For very low μ values, i.e., $D\ll z_{F}$, the probability for a domain to undergo more than one event can be neglected, i.e., each domain only suffers either zero or one events. Then, $z=qz_{1}$, where q is a random variable allowed to take only 0 or 1 as value. The variable q follows a Bernoulli distribution with mean $\langle q\rangle =\phi_{1}$ and variance $V_{q}=\phi_{1}\left(1-\phi_{1}\right)$, being $\phi_{1}$ the probability for a domain to undergo a single event, which coincides with μ, i.e., the mean number of events in a domain. Averaging Equation (1) among all the domains, then

(2) $$\langle L_{d}\rangle =A\langle g\rangle \langle q\rangle \langle z_{1}\rangle +B\langle g^{2}\rangle \langle q^{2}\rangle \langle z_{1}^{2}\rangle =\left(A\langle g\rangle +B\langle g^{2}\rangle \frac{\bar{z_{1}^{2}}}{z_{F}}\right)\phi_{1}z_{F}=\left(A\langle g\rangle +B\langle g^{2}\rangle z_{D}\right)D$$

where we have used the identities $\langle q^{2}\rangle =V_{q}+\langle q^{2}\rangle$ and $\langle z_{q}^{2}\rangle =\bar{z_{1}^{2}}$. If $N_{d}$ is the number of domains contained in a cell nucleus, then the mean number of lethal lesions in the cell nucleus is given by $\langle L_{n}\rangle =N_{d}\langle L_{d}\rangle =\left(\alpha_{0}+\beta_{0}z_{D}\right)D$, where $\alpha_{0}=N_{d}A\langle g\rangle$ and $\beta_{0}=N_{d}B\langle g^{2}\rangle$. The number of lethal lesions among cells is Poisson distributed and therefore the survival fraction is given by $S=\exp\left[-(\alpha_{0}+\beta_{0}z_{D})D\right].$ Interestingly, under these circumstances ($D\ll {\bar{z}}_{F}$), no quadratic term in the exponential for the survival fraction is predicted.

A more general result can be derived following the same reasoning. Let us consider a domain receiving exactly i events. Then, the specific energy imparted to that domain is a stochastic quantity given by $z=\sum_{i=1}^{\infty }q_{i}z_{q,i}$, where $q_{i}$ is a random variable among domains, which can only take the values 0 or i for a given domain; and $z_{q,i}$ is a random variable representing the specific energy imparted per event for a domain with exactly i events. Its average is again given by $\langle z_{q,i}\rangle =z_{F}$ and its variance is $V_{z_{1}}/i$. Again, each $q_{i}$ variable follows a Bernoulli distribution with mean $\langle q_{i}\rangle =i\phi_{i}$ and variance $\langle V_{q_{i}}\rangle =i^{2}\phi_{i}\left(1-\phi_{i}\right)$, so that $\langle q_{i}^{2}\rangle =i^{2}\phi_{i}$, where $\phi_{i}$ is the probability for a domain to receive exactly i events. Note that the macroscopic dose is then given by $D=\sum_{i=1}^{\infty }i\phi_{i}z_{F}$. Equation (2) now becomes

(3) $$\langle L_{d}\rangle =A\langle g\rangle \langle \overset{\infty }{\underset{i=1}{\sum }}q_{i}z_{q,i}\rangle +B\langle g^{2}{\rangle \langle \overset{\infty }{\underset{i=1}{\sum }}q_{i}z_{q,i}\rangle }^{2}=A\langle g\rangle \;\overset{\infty }{\underset{i=1}{\sum }}i\phi_{i}z_{F}+B\langle g^{2}\rangle \overset{\infty }{\underset{i=0}{\sum }}i^{2}\phi_{i}\left(z_{F}^{2}+\frac{V_{z_{1}}}{i}\right)$$

Equation (3) can be rewritten as

(4) $$\langle L_{d}\rangle =\left(A\langle g\rangle +B\langle g^{2}\rangle \frac{z_{F}^{2}+V_{z_{1}}}{z_{F}}\right)\overset{\infty }{\underset{i=1}{\sum }}i\phi_{i}z_{F}+\overset{\infty }{\underset{i=1}{\sum }}\left(i^{2}-i\right)\phi_{i}z_{F}^{2}=\left(A\langle g\rangle +B\langle g^{2}\rangle z_{D}\right)D+B\langle g^{2}\rangle \frac{\sum_{i=1}^{\infty }\left(i^{2}-i\right)\phi_{i}}{{\left(\sum_{i=1}^{\infty }i\phi_{i}\right)}^{2}}D^{2}$$

The number of events occurring in a domain follows, in general, a Binomial distribution. Let p be the probability for a potential event to happen, i.e., the individual probability for a particle track to interact with a domain. Then, after n tracks, the number of events in a domain has mean np and variance np(1−p). As $\phi_{i}$ represents the probability for a domain to suffer exactly i events, $\sum_{i}i\phi_{i}=np$ represents the mean of this Binomial distribution. In the same way, $\sum_{i}i^{2}\phi_{i}=np\left(1-p\right)+{\left(np\right)}^{2}$. Therefore, the quotient in Equation (4) becomes

(5) $$\frac{\sum_{i}\left(i^{2}-i\right)\phi_{i}}{{\left(\sum_{i}i\phi_{i}\right)}^{2}}=1-\frac{1}{n}$$

This result exactly coincides with Equation (2) when n=1, so that none of the domains can undergo more than one event per dose unit. In a similar way to the domain case, the mean specific energy imparted in an event to the nucleus is $z_{F,n}$ and the dose-mean specific energy is $z_{D,n}$. Let N be the number of tracks of incident radiation crossing the nucleus. Then, the dose imparted to the nucleus is given by $D=Nz_{F,n}$. The number of tracks crossing the nucleus, in turn, represents the maximum number of events for a domain: $n=N=D/z_{F,n}$. According to Equation (2), there is no quadratic term when $D\leq z_{F,n}$ (n≤1), while for $D>z_{F,n}$:

(6) $$\langle L_{d}\rangle =\left(A\langle g\rangle +B\langle g^{2}\rangle z_{D}\right)D+B\langle g^{2}\rangle \left(1-\frac{z_{F,n}}{D}\right)D^{2}=\left[A\langle g\rangle +B\langle g^{2}\rangle (z_{D}-z_{F.n})\right]\;D+B\langle g^{2}\rangle D^{2}$$

Independently, for very few particle tracks impacting the irradiated cells, lesions among cell nuclei can no longer be assumed as Poisson-distributed [35]. This effect occurs at doses even lower than the threshold here considered, as the scarceness of tracks crossing a single nucleus happens at higher doses than the scarceness of tracks among the set of cell nuclei. This effect has been demonstrated by Hawkins [17] to be characterized by including a saturation factor to account for the overkill effects for the alpha coefficient which is given by

(7) $$f_{s}=\frac{1-\exp\left[(-\left(\alpha_{0}+\beta_{0}z_{D}\right)z_{D,n}-\beta_{0}z_{D,n}^{2}\right]}{\left(\alpha_{0}+\beta_{0}z_{D}\right)z_{D,n}+\beta_{0}z_{D,n}^{2}}$$

For a higher dose so that the assumption of Poisson-distributed lesions among irradiated cell nuclei is valid, the survival fraction is a piecewise function, with a pure linear component for low doses and a linear quadratic from the threshold $D=z_{F,n}$:

(8) $$S\left(D\right)=\{\begin{array}{cc} \exp\left[-\left(\alpha_{0}+\beta_{0}z_{D}\right)\;f_{s}\;D\right] & \mathrm{if}\;D\leq z_{F,n}\\ S\left(z_{F,n}\right)\cdot \exp\left[-\left(\left(\alpha_{0}+\beta_{0}\left(z_{D}-z_{F,n}\right)\right)f_{s}\;\left(D-z_{F,n}\right)+\beta_{0}{\left(D-z_{F,n}\right)}^{2}\right)\right] & \mathrm{if}\;D>z_{F,n} \end{array}$$

where $S\left(z_{F,n}\right)=\exp\left[-\left(\alpha_{0}+\beta_{0}z_{D}\right)\;f_{s}\;z_{F,n}\right]$ is the survival fraction at the threshold dose $D=z_{F,n}$. Note that $z_{F,n}$ is a parameter characterized by the microdosimetric distribution of the incident radiation and the size of the cell nucleus. If the nucleus can be assumed to be a sphere of radius $r_{n}$, then $z_{F,n}$ can be expressed as

(9) $$z_{F,n}=\frac{y_{F}}{\rho \pi r_{n}^{2}}$$

where $y_{F}$ is the frequency-average lineal energy, whose value is equal to the LET of a monoenergetic particle and the track-average LET for a composed beam and ρ is the density of the nucleus, considered to be made of liquid water. Linear energy—and specific energy—can be determined experimentally [36,37] or from either time-consuming Monte Carlo (MC) simulations or analytical approaches, as our group has recently shown for protons [38] and alpha particles [39]. Hence, $z_{F,n}$ is proportional to the LET (or track-average LET) of the considered radiation. If $r_{n}$ is assumed to be 3.0 μm, which is the average nucleus radius for mammalian cells [40], this relation can be expressed by $z_{F,n}\left[\mathrm{Gy}\right]=0.0057\times \mathrm{LET}\left[\mathrm{keV}/\mu m\right]$. This assumption can be considered approximately valid up to around 200 keV/μm, since as LET further increases, sensitivity to different sizes of the nucleus plays a more important role [41]. For low-LET radiations, the initial linear part of S(D) can be neglected. However, as LET increases this initial linear region of S(D) becomes more relevant, which is in concordance with the literature [42].

### 2.2. Application to Mixed Radiations–Simultaneous Irradiations

Let a population of cells be simultaneously irradiated by two different types of radiations with high LET $L_{↑}$, imparting a dose $D_{↑}$, and low LET $L_{↓}$ imparting a dose $D_{↓}$, respectively. In the context of TDRA, it was shown [28,43] that such a combination of different radiations is characterized by a survival fraction

(10) $$S\left(D_{↑},D_{↓}\right)=\exp\left[-\left(\alpha_{↑}D_{↑}+\beta_{↑}D_{↑}^{2}+\alpha_{↓}D_{↓}+\beta_{↓}D_{↓}^{2}+2\sqrt{\beta_{↑}\beta_{↓}}D_{↑}D_{↓}\right)\right]$$

where, from the LQ survival curves for the high-LET and low-LET radiations, $\alpha_{↑}$ and $\alpha_{↓}$ are the linear parameters and $\beta_{↑}$ and $\beta_{↓}\;$are the quadratic parameters, respectively. This can be expressed as a single linear-quadratic expression, in which the parameters for the combination are given by

(11) $$\begin{array}{c} \alpha_{c}=\omega_{↑}\alpha_{↑}+\omega_{↓}\alpha_{↓}\\ \beta_{c}={\left(\omega_{↑}\sqrt{\beta_{↑}}+\omega_{↓}\sqrt{\beta_{↓}}\right)}^{2} \end{array}$$

where $\omega_{↑}$ and $\omega_{↓}$ are the fraction of dose for high- and low-LET radiations, respectively. As shown in Equation (8), for doses $D<z_{F,n}$, the quadratic parameter vanishes so that if both radiations were acting independently, Equation (10) would become

(12) $$S(D){|}_{\mathrm{ind}}=\{\begin{array}{ll} \exp\left[-\alpha_{c}D\right] & \mathrm{if}\;D\leq z_{F,n,↓}\\ S\left(z_{F,n,↓}\right)\exp\left[-\left(\alpha_{c}\left(D-z_{F,n,↓}\right)+\omega_{↓}^{2}\beta_{↓}{\left(D-z_{F,n,↓}\right)}^{2}\right)\right] & \mathrm{if}\;z_{F,n,↓}<D\leq z_{F,n,↑}\\ S\left(z_{F,n,↑}\right)\;\exp\left[-\left(\alpha_{c}\left(D-z_{F,n,↑}\right)+\beta_{c}{\left(D-z_{F,n,↑}\right)}^{2}\right)\right] & \mathrm{if}\;D>z_{F,n,↑} \end{array}$$

where $D=D_{↓}+D_{↑}$; $S\left(z_{F,n,↓}\right)=\exp\left(-\alpha_{c}z_{F,n,↓}\right)$; $S\left(z_{F,n,↑}\right)=S\left(z_{F,n,↓}\right)\exp\left[-\left(\alpha_{c}\left(z_{F,n,↑}-z_{F,n,↓}\right)+\beta_{↓}{\left(z_{F,n,↑}-z_{F,n,↓}\right)}^{2}\right)\right]$ and we have used the fact $z_{F,n,↓}<z_{F,n,↑}$. However, when a cell nucleus receives both radiations at the same time, for a given dose the number N of tracks crossing a cell nucleus is notably increased with respect to the same insult of only the high-LET radiation. As the number of tracks per unit dose is given by $1/z_{F,n}$, the combined mean specific energy imparted to the nucleus is given by

(13) $$z_{F,n,c}=\frac{1}{\frac{\omega_{↑}}{z_{F,n,↑}}+\frac{\omega_{↓}}{z_{F,n,↓}}\;}$$

and the survival fraction for the combination of radiations would be given by

(14) $$S\left(D\right){|}_{\mathrm{comb}}=\{\begin{array}{ll} \exp\left(-\alpha_{c}D\right) & \mathrm{if}\;D\leq z_{F,n,c}\\ S\left(z_{F,n,c}\right)\exp\left[-\left(\alpha_{c}\left(D-z_{F,n,c}\right)+\beta_{c}{\left(D-z_{F,n,c}\right)}^{2}\right)\right] & \mathrm{if}\;D>z_{F,n,c}, \end{array}$$

where $S\left(z_{F,n,c}\right)=\exp\left(-\alpha_{c}z_{F,n,c}\right)$. Note that if both weights are balanced and $z_{F,n,↓}\ll z_{F,n,↑}$ (or, equivalently, $L_{↓}\ll L_{↑}$), $z_{F,n,c}\approx z_{F,n,↓}$. This means that the linear part of the survival curve ends at much lower doses than $z_{F,n,↑}$, i.e., the action of the high-LET radiation acquires linear-quadratic nature before. In fact, a lesser survival fraction is predicted for the same dose from the combined effect given in Equation (14) than from the independent effect, given in Equation (13).

### 2.3. Sequential Irradiations

Operating on Equation (10), if two radiations are sequenced in an interval short enough to neglect any cellular repair, the $\alpha_{2,\mathrm{seq}}$ parameter of the second radiation in the sequence, after a dose $D_{1}$ from the first radiation, is enhanced according to

(15) $$\alpha_{2,\mathrm{seq}}=\alpha_{2}+2\sqrt{\beta_{1}\beta_{2}}D_{1}$$

where $\alpha_{2}$ and $\beta_{2}$ are the LQ parameters of the second radiation in the sequence and $\beta_{1}$ is the quadratic parameter of the first radiation in the sequence when acting alone. In a similar way to the previous reasoning, if a high-LET irradiation follows a low-LET irradiation, the initial linear part of the survival curve from Equation (8) can be also disregarded. Again, the low-LET radiation first produces a considerable number of events in each domain so that when the high-LET radiation begins, the distribution of events can be already considered Poisson-distributed. Thus, the quadratic component of the survival curve becomes prominent before with respect to the independent action assumption, in which $\beta_{↑}$ would be zero until a dose $z_{F,n,↑}$ was delivered.

However, the inverse order of the sequence (first high-LET and then low-LET radiations), would not avoid the linear section of the survival curve for the high-LET radiation, so no difference with respect to the independent action assumption would be expected.

### 2.4. Comparison to Published Experiments

Some published experiments for combined radiations with high LET and low LET were taken to assess the accuracy of our predictions. Table 1 shows the characteristic of each experiment: the type of radiations mixed, LET and corresponding $z_{F,n}$, whether they were simultaneous or sequential, cell line with the α and β parameters and their standard deviation for X-rays and the domain radius $r_{d}$ to compute $z_{D}$. In all experiments, $z_{F,n}=z_{D}=0$ for the X-ray radiation was considered. Although $r_{d}$ can be determined as a single value for a cell line from survival curves [44,45], we used a relation derived in a separate work [46] in which we related the domain radius with the X-ray ${\left(\alpha /\beta \right)}_{x}\;$ratio according to the relation

(16) $$r_{d}\;\left[\mu m\right]={\left(\frac{0.194}{{\left(\alpha /\beta \right)}_{x}\;\left[\mathrm{Gy}\right]}\right)}^{\frac{1}{3}}$$

Equation (16) assumes $r_{n}=$ 3.0 μm as mentioned before. However, for LET higher than approximately 100–200 keV/μm, the variability in the nucleus radius becomes relevant and this constant value can no longer be assumed. Consequently, for the Fe-56 case a different nucleus radius was obtained from the fit of the LQ model to the experimental data for Fe-56 irradiation alone, yielding $r_{n}=$ 4.5 μm, which is in agreement with values published in the literature [47].

Results using the original formulation of the MKM are also included for individual expositions of high-LET radiations, in order to assess the effects on survival curves of the modification for low doses proposed in this work. Values of the coefficient of determination, R^2^, are calculated for all curves to evaluate the agreement to the published experimental data.

## 3. Results

**Figure 1:**
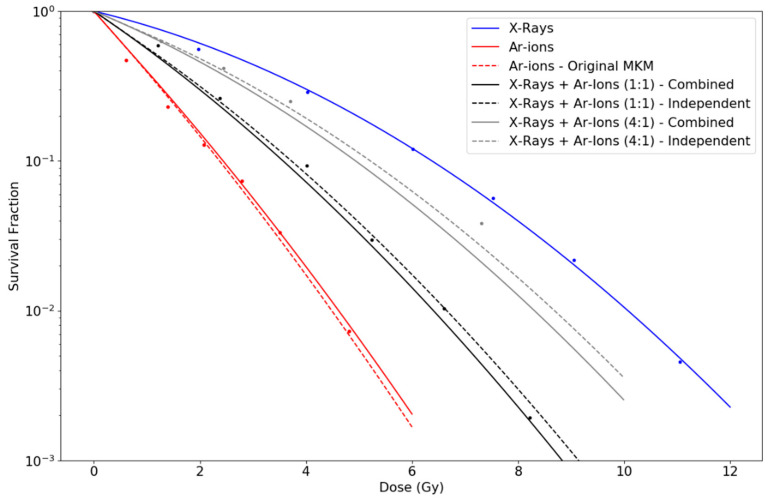
Survival fraction curves for simultaneous expositions of V79 cells to Argon ions and X-rays, as well as alone expositions, as done in Furusawa et al. [23]. LET for Argon ions is estimated to be 86 keV/μm. Simultaneous expositions were performed considering two different proportions: (1:1), which means equal contribution from each type of radiation; and (4:1), i.e., dose from X-rays was four times greater than dose from Argon ions. Blue curve shows the fit of the LQ model to the experimental data points for X-ray irradiation alone. The rest of the curves are calculated according to Equations (8), (10) and (12), respectively, with parameters $r_{n}=$ 3.0 μm and $r_{d}=$ 0.256 μm.

**Figure 2:**
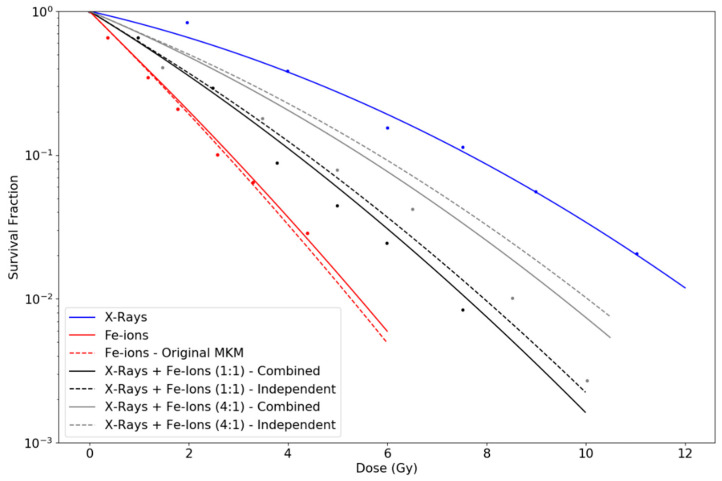
Survival fraction curves for simultaneous expositions of V79 cells to Iron ions, with LET considered to be 442 keV/μm, and X-rays and independent expositions, as done in Furusawa et al. [23]. As in Figure 1, two different proportions are considered: (1:1) and (4:1), respectively. The curve for X-rays shows a linear fit of the LQ model to the experimental data points. The rest of the curves are calculated according to Equations (8), (10) and (12), respectively, using the parameters $r_{n}=$ 4.5 μm and $r_{d}=$ 0.295 μm.

Figure 1 and Figure 2 show survival curves of V79 cells simultaneously exposed to Argon ions (86 keV/μm) and/or X-rays, as well as Fe-56 (442 keV/μm) and/or X-rays, respectively, by Furusawa et al. [23]. Data points reflect the experimental results published in that work. The curve for X-rays survival fraction is obtained by linearly fitting the LQ model to the experimental points. For the experiment in Figure 1, we obtained $\alpha_{0}=$ 0.195 Gy^−1^ and $\beta_{0}=$ 0.026 Gy^−2^. R^2^ values were found to be: 0.996 for X-rays; 0.98 for Ar-ions (for both modified and original MKM); 0.98 and 0.991 for the combined and independent action in 1:1 the mixture, respectively; and 0.991 and 0.997 for the combined and independent action in the 4:1 mixture, respectively.

Similarly, the parameters of the LQ from the fit to the experimental points for X-rays in Figure 2 were $\alpha_{0}=$ 0.181 Gy^−1^ and $\beta_{0}=$ 0.016 Gy^−2^. These values were used, respectively, to obtain the survival fraction for the simple irradiations with Argon and Iron ions according to Equation (8) and the survival fractions corresponding to independent and combined action using Equations (10) and (12), respectively. In this case, R^2^ values were 0.97 for X-rays; 0.98 for both curves of individual Fe-ions; 0.996 and 0.994 for the combined and independent action in the 1:1 mixture, respectively; and 0.95 and 0.93 for the combined and independent action in the 4:1 mixture, respectively.

**Figure 3:**
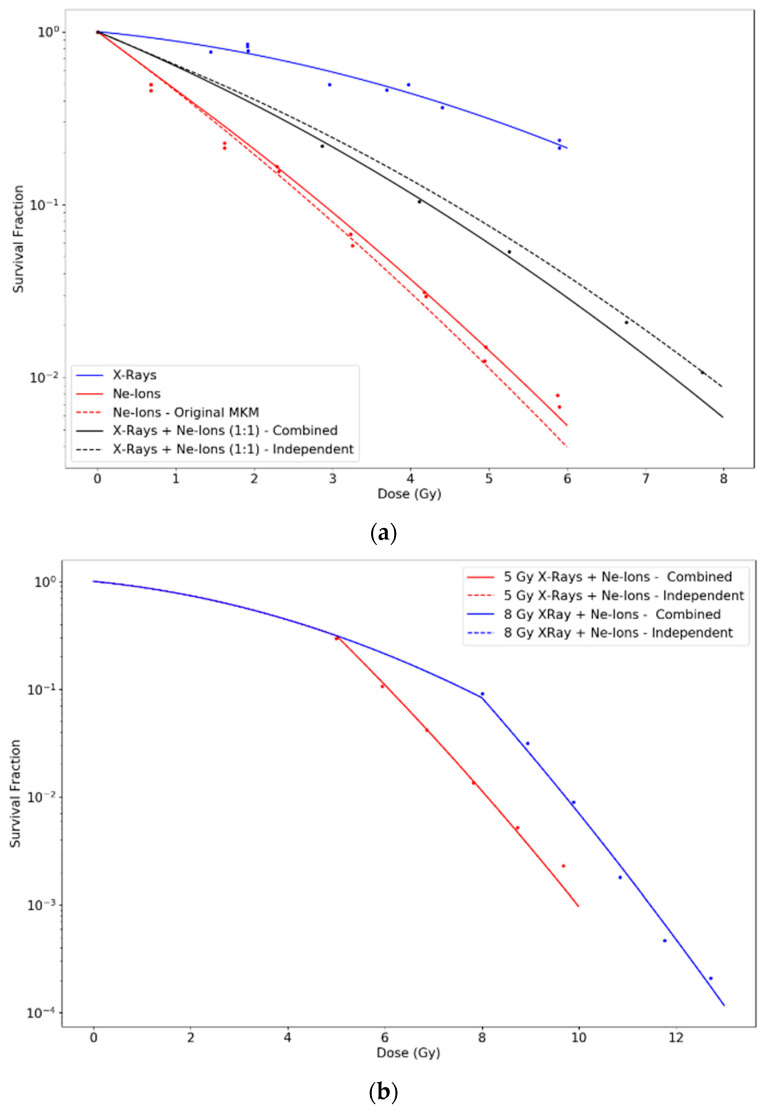
Survival curves for (**a**) X-rays, Neon ions and simultaneous irradiations in equal proportions; and (**b**) sequential irradiations of initial 5 Gy and 8 Gy of X-rays followed by insults of Neon ions, as done in Ngo et al. [25]. The blue curve in panel (a) was obtained by fitting the LQ model to the experimental points. The rest of the curves in both panels are obtained using those parameters and Equations (8), (10), (12) and (15), together with values $r_{n}=$ 3.0 μm and $r_{d}=$ 0.371 μm.

Figure 3 shows the results of our calculations for simultaneous and sequential exposures of V79 cells to X-rays and Neon ions with LET 183 keV/μm, taking experimental data from Ngo et al. [25]. For X-rays, the fit of the LQ model provided values $\alpha_{0}=$ 0.10 Gy^−1^ and $\beta_{0}=$ 0.026 Gy^−2^. Sequential irradiations were done with low-LET radiation first and high-LET radiation afterwards. The dose first deposited by low-LET radiation allows to disregard the initial linear portion of the high-LET survival curve, yielding the solid lines in Figure 3. Dashed lines represent the scenario in which the linear portion was not neglected. R^2^ values were 0.96 for X-rays; 0.97 for Ne-ions, calculated with both modified and original MKM; 0.9996 for the combined action in the 1:1 mixture and 0.996 for the independent action in the 1:1 mixture.

**Figure 4:**
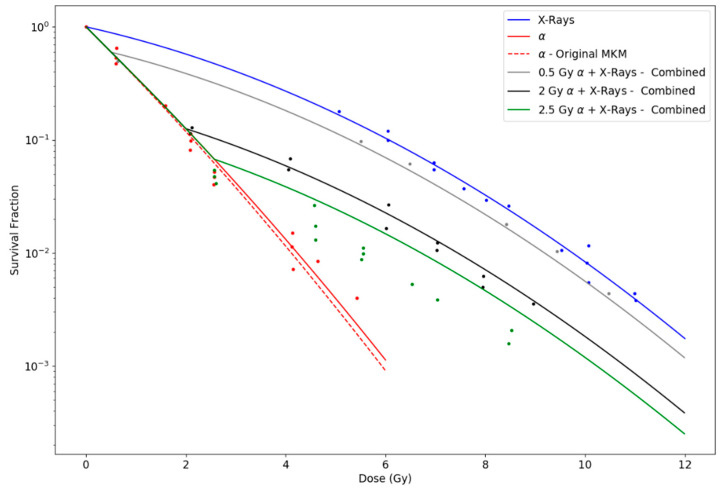
Survival curves for individual irradiations of X-rays and alpha particles with LET equal to 140 keV/μm, and for sequential irradiations of 0.5 Gy, 2.0 Gy and 2.5 Gy of the same alpha particles followed by X-rays, as done in McNally et al. [30]. Survival curve for X-rays was obtained by fitting the LQ model to the experimental points. The curve for alpha particles was obtained by using Equation (8) with parameters $r_{n}=$ 3.0 μm and $r_{d}=$ 0.279 μm, while survival curves for sequential exposures were obtained using Equation (15).

Finally, Figure 4 shows the results for the experiments from McNally et al. [30], in which slow alpha particles with LET 140 keV/μm and X-rays are used to irradiate V79 cells separately and in sequences. As high-LET radiation is done first, no independent action is represented in this case. LQ parameters for X-ray survival curve were obtained as $\alpha_{0}=$ 0.226 Gy^−1^ and $\beta_{0}=$ 0.025 Gy^−2^. Calculated R^2^ values were 0.9993 for X-rays; 0.98 for both curves of α particles alone; 0.995 for the 0.5 Gy (α) + X-rays curve; 0.96 for the 2 Gy (α) + X-rays curve; and 0.95 for the 2.5 Gy (α) + X-rays curve.

## 4. Discussion

A reformulation of the LQ model is presented in this work, introducing a linear expression at low doses. The threshold for low doses is influenced by the quality of the radiation, and also by the size of the nuclei of the irradiated cells, condensed in the concept of mean single-event specific energy imparted to the nucleus, $z_{F,n}$. As Equation (9) shows, this threshold linearly increases with the LET of the radiation, or track-average LET in the case of composed beams and decreases with the square of the cell nucleus radius. This initial linear part of the survival curve, therefore, might be disregarded for radiations of very low LET, particularly for X-rays, and would have more impact the smaller the cell nuclei is.

Indeed, under the theory of MKM, two different parameters related to the cell characteristics have an impact on the survival curve for a given cell line: not only the aforementioned nucleus radius, but also the domain radius, whose value has an analogous role in $z_{D}$. As pointed out by Kase et al. [41], for a fixed value of the nucleus radius, the different domain radii imply variabilities on the survival curves for low-LET radiations, but its effect tends to fade out for high-LET radiations. In contrast, provided a domain radius, the nucleus radius does not seem to influence survival curves for low-LET radiations, but definitely conditions the survival curves of high-LET radiations. The turning point between these two regions is here considered to be around 100–200 keV/μm, which approximately coincides with the saturation parameter to express the overkill effect ($y_{0}$ = 150 keV/μm). Note that, in contrast with the models by Kase et al. [37] and the Double Stochastic Microdosimetric Kinetic (DSMK) model proposed by Sato and Furusawa [19], our model does not rely on saturation-corrected parameters, and therefore does not need the knowledge of the probability density of specific energy in the domain and/or the nucleus. In fact, it only depends on the dose-mean specific energies, which can be calculated analytically as we have shown in previous works [5,38,39,48].

We have previously derived a function for the domain radius depending on the ${\left(\alpha /\beta \right)}_{x}$ ratio of the cell line, under the assumption of constant nucleus radius equal to 3.0 μm. Consistently, for radiations below 200 keV/μm, survival curves show agreement with the prediction from the MKM using a fixed nucleus radius but a variable domain radius from Equation (16). However, for radiations whose LET is largely beyond 200 keV/μm, as in the Fe-ions case, the combination of constant nucleus radius and domain radius given by Equation (16) is no longer valid. As discussed in other works, both parameters may vary among stages of cell cycle, oxygenation conditions and other variables [46,47]. This is underscored by this work, since although the same cell line is used in all the considered experiments, large variabilities are found in the ${\left(\alpha /\beta \right)}_{x}$ ratios and thus in the domain radii. Therefore, more research is needed to determine the interdependencies between these factors and the values of $z_{F,n}$ and $z_{D}$, since, as shown in this work, both play a relevant role in the actual effects of ion radiations, including protons.

Otherwise, using the parameters proposed for each experiment, the MKM model with an initial linear part is capable of reproducing the observed curves for individual irradiations of ions with diverse LET. The original formulation of the MKM results in a LQ model along the whole range of dose with a constant value of the quadratic parameter (β), although experimental observations of survival curves for cells exposed to high-LET radiations seem to contradict this, pointing towards β→0. However, if β→0 is assumed when combining radiations of different LET, the general trend observed is that cell survival tends to be overestimated with respect to experimental data. In contrast, our modified model predicts an initial null value for β for individual high-LET radiations, but a greater effective value when combining radiations of different LETs, which explains both scenarios. However, differences are more appreciable when considering mixtures of high- and low-LET radiations. Note that as doses increase, our proposed modification tends to approach the original MKM predictions. We calculated survival curves under two possible assumptions: (i) independent action, i.e., the initial linear part for the high-LET radiation occurs regardless the mixture; and (ii) combined action, i.e., the initial linear part vanishes when mixing the high-LET radiation with low-LET radiation. To this respect, Figure 2 and Figure 3, representing experiments using Fe-ions and Ne-ions, show a slightly better agreement for the combined action assumption, especially for the sequential irradiation of X-rays + Ne-ions in Figure 3b. However, Figure 1, in which Ar-ions are used, seems to indicate the opposite. In any case, differences between the two proposed alternatives depend on the extent of the initial linear part, which, as discussed, depends on the LET of the radiation and the cell nucleus radius. Finally, for the sequence of high-LET radiation plus low-LET radiation, the initial linear part cannot be disregarded. Figure 4 shows discrepancies between predictions in these cases using different initial doses of α particles followed by X-rays. In any case, the large number of uncertainties and variabilities among experiments, such as standard deviation in the LQ parameters, dose rate, environmental conditions, time after irradiation, determination of LET values, etc. requires the results shown in this work to be further validated with dedicated experiments.

In space, astronauts are exposed to multiple high-LET radiations simultaneously and the formalism presented here can be employed to understand potential biological effects from exposure. The combination of several low-dose expositions would contribute to increase the total number of radiation tracks crossing cell nuclei. This is particularly relevant when considering low-LET radiations, such as X-rays, gamma rays or fast protons, that may contribute with a considerable number of tracks, provoking Poisson-distributed events in each domain, and anticipating the linear-quadratic part of the survival curve. In other words, under these conditions, the complexity of radiation-induced damage to DNA-based domains may be enhanced with respect to the independent action assumption. In this work, as in the original formulation of the MKM, only targeted effects (TEs) are considered, i.e., effects produced by radiation directly hitting the cell in consideration. However, low-dose effects such as hyper radiosensitivity (HRS) or increased radioresistance (IRR) are not considered [49,50]. Similarly, non-targeted effects (NTEs), led by signaling effects from irradiated to non-irradiated cells, also play a role, especially at low doses. The lack of consideration of HRS and NTEs in the MKM may explain why experimental points for low dose are below than predicted MKM-based curves. The inclusion of NTEs in a framework together with the MKM has been already done in the so-called Integrated Microdosimetric Kinetic Model (IMKM) by Matsuya et al. [20]. The harmonization of these low-dose effects with our modified MKM remains as a potential improvement of this work. The saturation of the repair mechanisms at high doses [9] is neither considered in the MKM nor our proposed modification and still may be included as a future improvement of this work. Finally, at low dose rates, inverse-dose-rate effects might also be of importance in biological effects predictions [51].

## 5. Conclusions

Under the assumptions of the MKM, the LQ model for the survival curves of high LET is no longer valid when the number of tracks interacting with domains is low so that Poisson distribution of events cannot be assumed. This is translated into a model composed of an initial linear section, up to a dose equal to the mean specific energy imparted to the cell nucleus, $z_{F,n}$, followed by a linear-quadratic part. When combining these high-LET radiations with low-LET radiations, either simultaneously or sequentially beginning with the low-LET radiation, the number of events per unit dose quickly grows and events of radiation in a domain are Poisson-distributed. Hence, the linear part predicted for individual high-LET radiations vanishes and a synergistic effect is expected. The experimental data analyzed here partially validate these hypotheses and more experiments are required in conditions to exploit the effects discussed in this work.

## Figures and Tables

**Table 1:** List of published experiments analyzed in this work. Although all experiments were carried out on V79 cells, we took the α and β parameters from each separated experiment by fitting the linear quadratic (LQ) model to the X-ray survival curve in order to account for possible variabilities in experimental conditions. Sequential exposures were made in time intervals short enough not to lead to significant repairs (“short” time according to Ngo et al., and no more than 3–4 min according to McNally et al.). Dose rates reported in each article were: (a) for Furusawa et al.: 1 Gy/min for all radiations; (b) for Ngo et al.: 2.7 Gy/min for X-ray and 5 to 6 Gy/min for Ne-ions, respectively; and (c) for McNally et al.: 3 Gy/min for X-ray and 0.35 Gy/min for α particles. Uncertainties show the standard deviation of the fitted parameters. $r_{d}$ was obtained according to Equation (16), which, in turn, assumes a constant nucleus radius of 3.0 μm. However, for radiations of Linear energy transfer (LET) above approximately 100–200 keV/μm, the variability on the nucleus radius alters the saturation factor shown in Equation (7). Therefore, for the Fe-56 experiment, a different nucleus radius $r_{n}=$ 4.5 μm was obtained from fitting the LQ model to the experimental data. * Because of this, the relation $z_{F,n}\left[\mathrm{Gy}\right]=0.0057\;\mathrm{LET}\left[\mathrm{keV}/\mu m\right]\;$ is no longer valid.

Publication	Rads	$L_{↑}\;(\mathrm{keV}/\mu m)$	$z_{F,n}\;(\mathrm{Gy})$	Mix Type	$\alpha_{x}\;({\mathrm{Gy}}^{-1})$	$\beta_{x}\;({\mathrm{Gy}}^{-2})$	$r_{d}\;(\mu m)$
Furusawa, 2002 [23]	Ar + X-ray	86	0.49	Simultaneous	0.195 ± 0.016	0.0260 ± 0.0018	0.295
	Fe-56 + X-ray	442	1.11 *		0.18 ± 0.03	0.016 ± 0.004	0.256
Ngo, 1981 [25]	Ne-10 + X-ray	183	1.04	Both Seq X→Ne	0.10 ± 0.03	0.026 ± 0.006	0.371
McNally, 1988 [30]	α + X-ray	140	0.80	Seq α→X	0.23 ± 0.03	0.025 ± 0.003	0.279
